# Machine Learning-Based Nicotine Addiction Prediction Models for Youth E-Cigarette and Waterpipe (Hookah) Users

**DOI:** 10.3390/jcm10050972

**Published:** 2021-03-02

**Authors:** Jeeyae Choi, Hee-Tae Jung, Anastasiya Ferrell, Seoyoon Woo, Linda Haddad

**Affiliations:** 1 School of Nursing, University of North Carolina, Wilmington, NC 28403, USA; ferrella@uncw.edu (A.F.); woos@uncw.edu (S.W.); haddadl@uncw.edu (L.H.); 2 College of Information and Computer Science, University of Massachusetts, Amherst, MA 01002, USA; heetae@umass.edu

**Keywords:** smoking, machine learning, nicotine addiction, youth e-cigarette use, youth waterpipe use

## Abstract

Despite the harmful effect on health, e-cigarette and hookah smoking in youth in the U.S. has increased. Developing tailored e-cigarette and hookah cessation programs for youth is imperative. The aim of this study was to identify predictor variables such as social, mental, and environmental determinants that cause nicotine addiction in youth e-cigarette or hookah users and build nicotine addiction prediction models using machine learning algorithms. A total of 6511 participants were identified as ever having used e-cigarettes or hookah from the National Youth Tobacco Survey (2019) datasets. Prediction models were built by Random Forest with ReliefF and Least Absolute Shrinkage and Selection Operator (LASSO). ReliefF identified important predictor variables, and the Davies–Bouldin clustering evaluation index selected the optimal number of predictors for Random Forest. A total of 193 predictor variables were included in the final analysis. Performance of prediction models was measured by Root Mean Square Error (RMSE) and Confusion Matrix. The results suggested high performance of prediction. Identified predictor variables were aligned with previous research. The noble predictors found, such as ‘witnessed e-cigarette use in their household’ and ‘perception of their tobacco use’, could be used in public awareness or targeted e-cigarette and hookah youth education and for policymakers.

## 1. Introduction

Electronic cigarette (e-cigarette) use in youth in the U.S. has increased rapidly in the past ten years. The U.S. Surgeon General and the FDA Commissioner have declared it as an epidemic [1,2]. A survey conducted between 2014 and 2018 showed the rate of e-cigarette use in young adults (18–24 years) to be 7.6%; this is interpreted as approximately one in five adolescents currently using e-cigarettes [3,4]. These numbers are truly alarming and tend to be higher in the state-level surveys. Nicotine in e-cigarettes is highly addictive [5]. Its addictive nature is reported to be as much as that of cocaine and heroin [6]. Most e-cigarettes and other vaping products contain some level of nicotine [7]. For example, individual pods of Juul, one of the most popular vaping products with youth, contain nicotine concentrations equivalent to a pack of cigarettes [8]. The earlier people start nicotine use, the harder it is to quit later in life. This is especially true for e-cigarette users under the age of 25 as their brains and nervous systems are not fully developed and are more vulnerable to nicotine addiction [9]. Recent studies have shown that in addition to highly toxic nicotine, vaping products also contain carcinogens, heavy metals, and other harmful substances [10].

While e-cigarettes and similar electronic nicotine delivery systems are promoted as less harmful than smoking tobacco products, they are far from harmless. Over 2600 lung-injury cases have been identified as E-Cigarette or Vaping Product Use-Associated Lung Injuries (also known as EVALI) in the U.S. (August 2019–January 2020) [11]. Of those cases, >15% were 17 years old or younger [12]. Vaping products and hookah smoking cause nicotine addiction in non-smokers and various health issues (e.g., may lead to cancer, changes in cardiac functions, respiratory problems) [13]. Ingestion or direct contact with these products is toxic and can be fatal [14,15]. 

E-cigarettes’ threat to health comes from their common ingredients (e.g., oxidizing agents, carcinogens, heavy metals) [16]. For starters, vegetable glycerol and propylene glycol, which are frequent additives in e-liquids, can create additional harmful chemicals—aldehydes—when heated [16]. Exposure to these chemicals can increase the gut microbiome’s vulnerability to leaking, leading to loss of essential molecules and microbes. Depletion of this microbiome can promote chronic inflammation, which in turn puts the body at risk of cancers and buildup of plaque around artery walls [17]. Eventually, this buildup can lead to angina, stroke, and myocardial infarction [18,19,20]. Although there is not enough evidence to demonstrate e-cigarettes’ direct and long-term damage to the cardiovascular system, e-cigarette use has been associated with increased heart rate, blood pressure, thrombosis, inflammation, oxidative stress, and vascular dysfunction [21,22]. Additionally, acrolein and carbonyls in e-liquids are high enough to lead to cardiovascular toxicity. Even if none of these solvents were present in e-liquids, nicotine by itself is known to increase cardiac arrhythmias [23]. E-cigarettes’ effect on pulmonary health reveals users’ further vulnerability from this product. 

Flavorings in e-cigarettes are toxic and can permanently damage human lungs, causing disbalance in the levels of the lungs’ inflammation proteins and the killing of lung cells altogether [24,25]. Heavy metals (e.g., nickel, lead, cadmium) in e-cigarette liquids are additional damaging irritants to the lungs and the airway overall. Although these metals are present in trace amounts, they can change human DNA and cause cell mutation, which in turn can lead to cancer [21]. Constituents of e-cigarette liquids such as pulegone, acrolein, formaldehyde, tobacco-specific nitrosamines, and oxidants can also raise the risk of cancer [21]. E-cigarettes have been on the U.S. market for a little over a decade [26]. This limits the amount of longitudinal data available to show the long-term effects of this product. However, the known effects of e-cigarette’s ingredients on their own should be sufficient to anticipate potential risks.

Recent studies show increased vulnerability to COVID-19 in adult and youth e-cigarette vapers and smokers [27,28,29]. Although there is no published evidence in the connection of hookah use and susceptibility to COVID-19, the social nature of this activity and the composition of a hookah device were noted by the World Health Organization (WHO) as potential risks of transmission [30]. While the evidence is still emerging on the health effects and safety of vaping, there is limited knowledge for e-cigarette and hookah cessation programs. Youth are often restricted from adult tobacco cessation programs or holistic interventions. In addition, prediction models based on identified predictor variables can be used for early intervention and prevention programs effectively. The aim of this study was to identify predictor variables such as social, mental, and environmental determinants that cause nicotine addiction in youth e-cigarette or hookah users and build nicotine addiction prediction models using machine learning algorithms. 

## 2. Materials and Methods

We applied machine learning, which is one of the data mining techniques to process and analyze data. We used the publicly available 2019 National Youth Tobacco Survey (NYTS) dataset collected electronically and selected e-cigarette or hookah users who had tried the products at least once in their lifetime. The mode imputation for missing data and two machine learning algorithms, Random Forest with ReliefF and Least Absolute Shrinkage and Selection Operator (LASSO), were applied to identify nicotine addiction predictor variables and build prediction models. MATLAB 2020b (Natick, MA, USA) was used for training models and 10-fold cross-validation for evaluating their performance. 

### 2.1. The National Youth Tobacco Survey (NYTS)

The NYTS was developed to collect necessary datasets for supporting the design, implementation, and evaluation of tobacco prevention and control programs nationally. This survey’s datasets are more comprehensive than those in the Youth Risk Behaviors Surveillance Systems, especially on tobacco-related indicators for middle- and high-school students. They include: (1) tobacco use (e.g., cigarettes, cigars, smokeless tobacco, electronic cigarettes, hookahs, roll-your-own cigarettes, pipes, snus, dissolvable tobacco, bidis, and heated tobacco products), (2) exposure to secondhand tobacco smoke and e-cigarette aerosol, (3) smoking cessation, (4) minors’ ability to purchase or obtain tobacco products, (5) knowledge and attitudes about tobacco, and (6) familiarity with pro-tobacco and anti-tobacco media messages [31]. The NYTS data were collected between 15 February 2019 and 24 May 2019. A total of 19,018 students completed the tablet-based or web-based questionnaires, yielding an 85.8% student response rate. The 2019 NYTS was reviewed and approved by the Office of Management and Budget, the contracted data collectors’ institutional review board (IRB), and the Center for Disease Control and Prevention (CDC)’s IRB. 

### 2.2. Data Preprocessing and Defining Labels

We removed irrelevant variables, such as student login and primary sampling unit numbers, and variables asking for the text form of answers (e.g., a brand name of cigarettes used). A total of 193 variables were included in the final analysis after one variable was used as a label—severity of nicotine addiction. 

The missing data rate of 27.0% was calculated based on missing data (*n* = 339,770) and total expected data (N = 1,256,623). We used mode imputation for missing data. The mode imputation is an easy-to-use and straightforward way to replace missing values by filling in the mode [32]. 

We used the time to the first cigarette of the day (TTFC) tool to interpret the severity of subjects’ nicotine dependence. TTFC is a strong predictor of quitting behavior and can independently predict the maintenance of quitting attempts by assessing the severity of nicotine addiction [33,34]. According to studies indicating “time to the first cigarette of the day”, the following nicotine dependence variables were labeled as (1) heavy nicotine addiction when a response is within 5–30 min, (2) moderate nicotine addiction when it is longer than 30 min to 1 h, (3) light nicotine addiction when it is longer than 1 h but less than 24 h, and (4) non-nicotine addiction when a response is either no use of cigarette or using it rarely [33,35,36,37,38].

### 2.3. Machine Learning Algorithms and Feature Selection

#### 2.3.1. Least Absolute Shrinkage and Selection Operator (LASSO)

The LASSO is a regression analysis method. It selects variables and performs regularization to increase the prediction accuracy [39]. There are many advantages of using LASSO, especially as it excludes irrelevant variables to improve interpretation of the prediction models [40]. We applied LASSO to build the prediction models since it has been used for various types of data including from surveys [41,42]. 

#### 2.3.2. Random Forest with ReliefF Variable Selection 

Random Forest is an ensemble algorithm that has a computational efficiency over larger datasets. This algorithm randomly selects a subset of variables and constructs many decision trees. Strengths of Random Forest are low bias, high variance, and low correlation between constructed trees [43,44,45]. Sixty-four decision trees were set in Random Forest, which was recommended in a study as the optimal number of trees [45].

The ReliefF method is an enhancement of the Relief algorithm, which is one of the best-known variable selection methods. Relief makes highly accurate and effective variable estimation. However, it shows low performance on incomplete data since its best estimation is attained through weighting. ReliefF is enhanced to overcome these limitations [46]. We used ReliefF to select variables and the Davies–Bouldin clustering evaluation index [47] to decide the optimal number of variables. Then, the Random Forest algorithm was applied to build the prediction models with the optimal numbers of variables. 

### 2.4. Evaluation of Performance of Prediction Models 

Root Mean Square Error (RMSE) is a standard way of measuring the errors of prediction models, and the Confusion Matrix is a specific table describing the performance of a classification model. We computed the RMSE to evaluate the performance of the prediction models and visualize the performace of developed prediction models in the Confusion Matrix. 

## 3. Results

A total of 6511 participants (mean age = 15.36 ± 1.85 years old) were identified as ever being e-cigarette or hookah users. Most of them were non-Hispanic (*n* = 4540, 69.73%) and white (*n* = 4504, 69.18%). They lived with someone who smokes cigarettes (*n* = 2131, 32.73%), e-cigarettes (*n* = 1560, 23.96%), or hookahs (*n* = 256, 3.92%). Two-thirds of participants indicated English as their first language (*n* = 4244, 65.18%) and did not have any disability (*n* = 4384, 74.24%). Table 1 shows the characteristics of participants in this study. 

### 3.1. Evaluation of Performace of the Prediction Model

**Figure 1:**
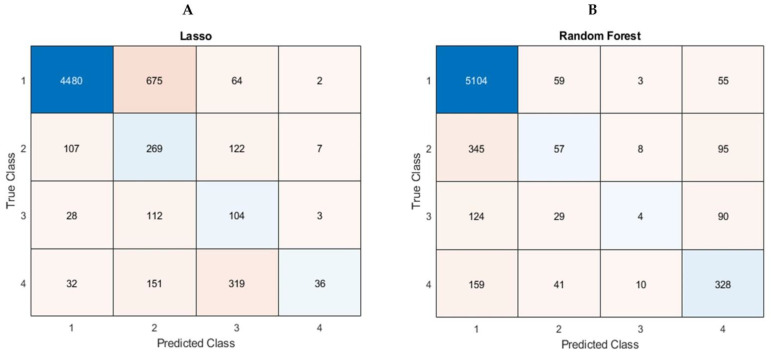
Confusion Matrices from LASSO (**A**) and Random Forest (**B**). Class 1 is a non-nicotine addicted group, Class 2 a lightly addicted group, Class 3 a moderately addicted group, and Class 4 a heavily addicted group. Note: Diagonal numbers from top left to bottom right are the numbers the model correctly identified a class.

Table 2 is a summary of performance of prediction, and Figure 1 shows the Confusion Matrices of LASSO and Random Forest. RMSE was the average RMSE from ten trained prediction models. Accuracy was calculated using data in each Confusion Matrix. All correctly predicted numbers in diagonal cells were summed and divided by the total number of participants (N = 6511). 

Table 3 is a summary of accuracy comparison based on Confusion Matrices in classes (different severity of nicotine addiction groups). Since numbers in diagonal cells in Confusion Matrices indicate correct predictions in each class, we tabulated them for accuracy comparison between two machine learning algorithms. For example, we simply divided correct prediction numbers in Class 1 (*n* = 4480) by the total number of participants (N = 6511). 

### 3.2. Predictor Variables Used in the Nicotine Addiction Prediction Models

LASSO and Random Forest identified an average of 85 and 41 predictor variables to build the prediction models, respectively. All 41 predictor variables except one were also identified by LASSO. Table 4 shows the 40 predictor variables identified by both LASSO and Random Forest. 

## 4. Discussion

We identified predictor variables and built the nicotine addiction prediction models using machine learning algorithms for youth e-cigarette or hookah users. The models built by LASSO and Random Forest (RF) with ReliefF show high predictive performance. RMSE of both models were almost the same (LASSO: 0.7509 vs. RF: 0.7436). Since there are no standard ranges of good RMSE values, it was defined based on the study sample size (N = 6511) and closer to zero. Thus, we interpreted that the RMSE values indicated high performance of prediction. Accuracy of the models by RF (0.7342) was higher than that of by LASSO (0.6370) overall. However, the models from each machine learning algorithm showed different strengths of prediction performance. The Confusion Matrix of LASSO showed a higher performance on predicting Class 2 (a lightly addicted group) and Class 3 (a moderately addicted group) than RF. Meanwhile, the Confusion Matrix of RF showed a higher performance on predicting Class 1 (a non-nicotine addicted group) and Class 4 (a heavily addicted group) than LASSO. 

### 4.1. Predictor Variables Aligned with the Literature

The predictor variables identified by both machine learning algorithms confirmed many socio-environmental determinants, such as peer pressure and brand name of tobacco products [48,49,50,51], as important predictor variables. Studies indicate there are several factors closely associated with youth nicotine addiction, including age of starting tobacco use, gender, grade level, ethnicity/race, living with current tobacco users, English not being the first language at home, and having a disability/mental health condition [42,52,53,54,55,56,57,58]. The results show that LASSO’s 85 predictor variables include age of starting tobacco use, gender, grade level, English not being the first language at home, and having a disability or mental condition. However, RF only includes age of starting tobacco use and having a disability or mental health condition in its 41 predictor variables. Although current studies indicate that living with current tobacco users and English not being the first language at home are predictor variables [56,58], both LASSO and RF-based prediction models do not use them in the models. These may be justified by half the survey participants not living with smokers and two-thirds of them speaking English as their first language at home. 

### 4.2. Predictor Variables Identified in Detail 

Items 13 and 32 in Table 4 reflect the evidence that specific types and brands of e-cigarette products can determine higher nicotine dependence [59]. For example, pod-based e-cigarettes have been found to result in stronger nicotine addiction [60]. Compared to other tobacco products, e-cigarettes have a minimal requirement for a warning label [61]. Warnings of nicotine addiction on e-cigarette packaging can motivate users to quit [62] and it can be the case with government’s recommended labels. However, warning labels provided by tobacco companies, on the other hand, are shown to be harder to read and understand [63]. Therefore, item 38 in Table 4 reflects the struggle between health warnings’ reach of e-cigarette users and participants’ motivation to quit this tobacco product. Alternatively, cues of a substance can trigger a person to use this substance. Response to these triggers can create a behavioral conditioning, which can lead to a reinforced nicotine addiction [64]. Therefore, when a participant frequently smells e-cigarette vapor (item 40), this person is likely to struggle avoiding e-cigarette use and is likely to progress to nicotine dependence. Overall, items listed in Table 4 provide helpful guidance in selection of variables for future research. 

### 4.3. Implication for Practice and Future Study

This study also confirms prior published evidence and will aid future investigators in a more educated use of variables. Tobacco companies are known to divide their specific product marketing to separate populations (e.g., menthol cigarettes, smokeless tobacco) [65,66]. E-cigarette marketing was long suspected of targeting white, middle-class, hipster youth and creating a separate culture of tobacco users [67,68]. Thus, it is not surprising that students who tried e-cigarettes at least once are predominately from white, non-Hispanic households that are either native to the U.S. or are acculturated to the English-speaking society. Although it was expected that e-cigarette use among youth is more prevalent in older adolescents based on prior 2011–2018 trends [3], what is most surprising is the similarity in the rates of e-cigarette experimentation among female and male students. Prior research shows e-cigarette use rates tend to be higher among self-identified male students than those who identify themselves as female [69]. Considering that exposure to e-cigarette marketing is significantly associated with adolescents’ ever and current e-cigarette use [70,71], this change in gender differences may be accounted to tobacco companies’ increased e-cigarette marketing [72]. Further research is needed to identify any connection between gender differences in e-cigarette marketing expenditures, adolescents’ exposure, and their e-cigarette use. Prior studies have demonstrated the role modeling of tobacco use among family on adolescents’ smoking and vaping [57,73]. Our results confirm that role modeling is still present among e-cigarette experimenters (e.g., more than half of these respondents (54.38%) reported living with a tobacco user). However, an interesting finding is that less than a quarter of participants witnessed e-cigarette use in their household (23.96%). This trend is worth investigating further. It would be important to distinguish whether participants’ experimentation with e-cigarettes occurs due to the role modeling of tobacco use in general or whether it is related to participants’ perception of their tobacco use as a safer or better alternative to that of their family members. Although e-cigarettes are promoted as safer tobacco alternatives, research has shown that e-cigarette use among youth is a gateway to smoking and can lead to higher frequency of smoking among youth [74,75]. 

### 4.4. Limitations and Strengths

We were able to build machine learning-based nicotine addiction prediction models for middle and high schoolers using publicly available survey data. Although the survey showed a good return rate and the size of data was large, there were still limitations. Data were self-reported; validity of data is contingent upon respondents’ honesty and memory. The degree of under-reporting and over-reporting of information was unknown. This survey was cross-sectional, measuring responses at a single snapshot in time, hence overlooking the development of nicotine addiction and dependency behaviors as they develop over time. If other variable selections and machine learning algorithms were used, there may have been different predictor variables showing different optimal performance. Our results were based on responses from ever e-cigarette and hookah users, collected prior to the federal Tobacco 21 legislation [76] as the data from the 2020 NYTS was not yet released. It is possible that responses collected post-December 2019 and those during the COVID-19 outbreak in the U.S. may further alter this list of predictors. Nevertheless, the major strength of the study results is the predictor variables identified by the nicotine addiction prediction models that could be used to design and develop nicotine addiction early intervention and prevention programs for youth.

## 5. Conclusions

We successfully used two machine learning algorithms (LASSO and RF with ReliefF) to identify nicotine addiction predictor values and built high-performing nicotine addiction prediction models for youth e-cigarette and Hookah users. 

Results of this study can guide the focus of future tobacco prevention programs and cessation studies. For example, participants’ perceptions about the dangers of e-cigarette use indicate the need to expand adolescents’ awareness about these products. Adolescents’ preference for menthol-containing products can be an opportunity to include these products in the awareness campaign. Age at tobacco initiation highlights the point at which or immediately before which it is crucial to introduce the tobacco prevention programs. Experimentation with heated tobacco products reveals adolescents’ continued fascination with novel tobacco products and new technology, which implies adolescents’ potential vulnerability to these products and the possibility of heated tobacco products’ displacement of currently popular electronic cigarettes. The addition of these products to prevention programs and their stricter control by the FDA may help prevent the perpetuation of the current e-cigarette epidemic among youth. 

Success and number of attempts in cessation highlight the need for the expansion and reconsideration of tobacco cessation help for underage groups. Adolescents’ access to tobacco products, their exposure to warning labels, and proximity to others using tobacco products show the importance of stricter tobacco control in these areas. Besides, results may be used in targeted e-cigarette and hookah youth education and by policymakers in kindergarten to 12th grade education.

## Figures and Tables

**Table 1:** Characteristics of study participants (N = 6511).

Variables/Categories		N (%)
**Age**	9	15 (0.23)
	10	1 (0.02)
	11	88 (1.35)
	12	379 (5.82)
	13	698 (10.72)
	14	932 (14.31)
	15	1078 (16.56)
	16	1190 (18.28)
	17	1323 (20.32)
	18	746 (11.46)
	19	56 (0.86)
	NA	5 (0.08)
**Grade**	6th	330 (5.07)
	7th	610 (9.37)
	8th	844 (12.96)
	9th	1058 (16.25)
	10th	1151 (17.68)
	11th	1242 (19.08)
	12th	1252 (19.23)
	NA	24 (0.37)
**Gender**	Male	3446 (52.93)
	Female	3042 (46.72)
	NA	23 (0.35)
**Ethnicity**	Non-Hispanic	4540 (69.73)
	Mexican	1052 (16.16)
	Puerto Rican	190 (2.92)
	Other Hispanic	90 (1.38)
**Race**	American Indian	554 (8.51)
	Asian	396 (6.08)
	Black	1194 (18.34)
	Hawaiian	261 (4.01)
	White	4504 (69.18)
**Lives with someone who uses**	Cigarettes	2131 (32.73)
	Cigars	580 (8.91)
	Chewing tobacco	697 (10.70)
	E-cigarettes	1560 (23.96)
	Hookahs	256 (3.92)
	Pipes	168 (2.58)
	Snus	117 (1.80)
	Dissolvable tobacco	105 (1.61)
	Bidis	98 (1.51)
	Heated tobacco	131 (2.01)
	No tobacco products	2964 (45.62)
**English is their first language**	Yes	4244 (65.18)
	No	2106 (32.35)
	NA	161 (2.47)
**Has disability**	Yes	1482 (22.76)
	No	4384 (74.24)
	NA	195 (2.99)

NA = Not answered.

**Table 2:** Performance of prediction.

Algorithm	Root Mean Square Error (RMSE) (SD)	Accuracy
LASSO	0.7509 (±0.0287)	0.6370
Random Forest	0.7436 (±0.0401)	0.7342

**Table 3:** Comparison of accuracy based on Confusion Matrices (N = 6511).

Class	LASSO (%)	Random Forest (%)
1: Non-nicotine addicted	4480 (68.81)	5104 (78.39)
2: Lightly addicted	269 (4.13)	57 (0.88)
3: Moderately addicted	104 (1.60)	4 (0.06)
4: Heavily addicted	36 (0.55)	328 (5.04)

**Table 4:** Predictor variables used both by LASSO and Random Forest.

	List of Predictor Variables Identified by both LASSO and Random Forest
1	How old were you when you first tried cigarette smoking, even one or two puffs?
2	How strongly do you agree with the statement ‘All tobacco products are dangerous’?
3	During the past 30 days, on how many days did you smoke cigarettes?
4	When was the last time you smoked a cigarette, even one or two puffs?
5	During the past 30 days, what brand of cigarettes did you usually smoke?
6	Menthol cigarettes are cigarettes that taste like mint. During the past 30 days, were the cigarettes that you usually smoked menthol?
7	How old were you when you first tried smoking a cigar, cigarillo, or little cigar, even one or two puffs?
8	During the past 30 days, on how many days did you smoke cigars, cigarillos, or little cigars?
9	Have you ever used chewing tobacco, snuff, or dip, such as Copenhagen, Grizzly, Skoal, or Longhorn, even just a small amount?
10	How old were you when you used chewing tobacco, snuff, or dip for the first time?
11	In total, on how many days have you used e-cigarettes in your entire life?
12	During the past 30 days, on how many days did you use e-cigarettes?
13	During the past 30 days, what brand of e-cigarettes did you usually use?
14	Have you ever tried a “heated tobacco product”, even just one time?
15	During the past 30 days, on how many days did you use any tobacco product(s)?
16	During the past 30 days, have you had a strong craving or felt like you really needed to use a tobacco product of any kind?
17	Are you seriously thinking about quitting the use of all tobacco products?
18	During the past 12 months, how many times have you stopped using all tobacco products for one day or longer because you were trying to quit all tobacco products for good?
19	Are you seriously thinking about quitting cigarettes?
20	During the past 12 months, how many times have you stopped smoking cigarettes for one day or longer because you were trying to quit smoking cigarettes for good?
21	During the past 30 days, did anyone refuse to sell you any tobacco products because of your age?
22	During the past 30 days, how often did you see a warning label on a cigar, cigarillo, or little cigar package?
23	During the past 30 days, how often did you see a warning label on a package of hookah tobacco?
24	How much do you think people harm themselves when they smoke cigarettes some days but not every day?
25	During the past 7 days, on how many days did someone smoke tobacco products in your home while you were there?
26	During the past 7 days, on how many days did you ride in a vehicle when someone was smoking a tobacco product?
27	Because of a physical, mental, or emotional condition, do you have serious difficulty concentrating, remembering, or making decisions?
28	During the past 30 days, on the days you smoked, about how many cigarettes did you smoke per day?
29	During the past 30 days, on the days that you smoked, about how many cigars, cigarillos, or little cigars did you smoke per day?
30	If one of your best friends were to offer you a cigar, cigarillo, or little cigar, would you smoke it?
31	During the past 30 days, on how many days did you use chewing tobacco, snuff, or dip?
32	Which of the following best describes the type of e-cigarette you have used in the past 30 days? If you have used more than one type, please think about the one you use most often.
33	How old were you when you first tried smoking tobacco in a hookah or waterpipe, even one or two puffs?
34	Do you think that you will try smoking tobacco in a hookah or waterpipe soon?
35	Do you think you will smoke tobacco in a hookah or waterpipe in the next year?
36	If one of your best friends were to offer you a hookah or waterpipe with tobacco, would you try it?
37	How easy do you think it is for people your age to buy tobacco products online?
38	During the past 30 days, how often did you see a warning label on an e-cigarette package?
39	How much do you think people harm themselves when they use chewing tobacco, snuff, dip, snus, or dissolvable tobacco products, some days but not every day?
40	During the past 30 days, on how many days did you smell the vapor from someone who was using an e-cigarette in an indoor or outdoor public place?

## Data Availability

Publicly available datasets were analyzed in this study. This data can be found here: https://www.cdc.gov/tobacco/data_statistics/surveys/nyts/index.htm (accessed on 22 January 2021).
